# Indoor Humidity

**Published:** 1904-12

**Authors:** 


### INDOOR HUMIDITY.

We have examined with unusual interest a copy of an article which Dr. Henry Mitchell Smith read before the Brooklyn Medical Society. Dr. Smith calls attention anew to the radical error in the arrangements for heating in common use to-day, namely, the absence of provision for supplying moisture needed in the air. He declares that in heating buildings we have failed to appreciate the full importance of the fact that our sensations of temperature depend not on the heat that is received from outside sources, but on the rapidity with which the body heat is lost. Radiation from the body is much more rapid when the air is lacking in moisture. To supply this moisture is a measure both hygienic and economic. On the latter point of view he quotes an authority as saying "twenty-five per cent. of the cost of heating is expended in raising the temperature from 60 to 70. So if we can keep comfortable at a temperature of 65, we shall have saved at least 12.5 per cent. of the total cost of heating." Relative to the above temperatures, Dr. Smith cites the results of an elaborate series of experiments, in which he found that a temperature of 65 degrees, when the air contained a proper amount of moisture, was warmer and more comfortable than a temperature of 72 or 74 degrees, with only half enough humidity. Dr. Smith has so repeatedly demonstrated this fact that he declares that it should be a cardinal rule that if a room at 68 is not warm enough for any healthy person it is because the relative humidity is too low, and in such case the procedure is to raise the relative humidity, not the temperature. Dr. Smith recommends that every household should have a hygrometer, and that water should be evaporated in rooms in sufficient amount to secure a relative humidity of about 60 per cent. In the absence of the hygrometer, a simple test will be to evaporate a sufficient amount of moisture to make the room comfortable at a temperature of 65 or 68 degrees. Dr. Smith suggests that the increase of catarrhal and other diseases in the winter time, including pneumonia, may be ascribed in some measure to the difference in the humidity between indoor and outdoor air. Not only is the dryness of the air conducive to hyperactivity of the glandular structures, but the passing from dry indoor air to moist outdoor air, and vice versa, is a severe strain on the mucous membranes. These facts are of great importance to the physician. For him to regulate the ventilating and heating apparatus of his patients' homes will be a tremendous task, and yet many affections can not be controlled until this important aid to health is properly looked after.—*Journal A. M. A.*

